# Cushing’s disease and negative MRI: a single-center series, systematic literature review, and meta-analysis

**DOI:** 10.1007/s10143-026-04168-2

**Published:** 2026-02-25

**Authors:** Francesca Ghidoni, Alba Madoglio, Sabrina Chiloiro, Lodovico Terzi di Bergamo, Marika Vezzoli, Simona Serioli, Giada Cosentino, Giorgio Saraceno, Isabella Lupi, Claudio Urbani, Massimo Licini, Renato Candrina, Antonella Giampietro, Ilenia Pirola, Carlo Cappelli, Davide Mattavelli, Vittorio Rampinelli, Marco Ferrari, Fausto Bogazzi, Mirco Cosottini, Roberto Gasparotti, Luca Manetti, Antonio Bianchi, Piero Nicolai, Cesare Piazza, Marco Maria Fontanella, Pietro Luigi Poliani, Francesco Doglietto

**Affiliations:** 1 Department of Translational Medicine and for Romagna, University of Ferrara, Ferrara, Italy; 2 Neurosurgery Unit, University Hospital of Ferrara, Ferrara, Italy; 3 Pituitary Unit, Department of Endocrinology and Diabetes, Fondazione Policlinico Universitario A. Gemelli, IRCCS, Rome, Italy; 4 Department of Medicine and Translational Surgery, Università Cattolica del Sacro Cuore, Rome, Italy; 5 Institute of Oncology Research, Bellinzona, Switzerland; 6 Biostatistics, Department of Molecular and Translational Medicine, University of Brescia, Brescia, Italy; 7 Department of Neurosurgery, Papa Giovanni XXIII, Bergamo, Italy; 8 Technology for Health PhD Program, University of Brescia, Brescia, 25124 Italy; 9 Department of Clinical and Experimental Medicine, University of Pisa, Pisa, Italy; 10 Neurosurgery, Department of Medical and Surgical Specialties, Radiological Sciences and Public Health, University of Brescia, Brescia, Italy; 11 Department of Clinical and Experimental Sciences, ASST Spedali Civili of Brescia, SSD Endocrinologia, University of Brescia, Brescia, Italy; 12 Endocrinology, Diabetology and Metabolic Diseases, Fondazione Poliambulanza Istituto Ospedaliero, Brescia, Italy; 13 Unit of Otorhinolaryngology-Head and Neck Surgery, Department of Surgical Specialties, Radiological Sciences and Public Health, ASST Spedali Civili of Brescia, University of Brescia, Brescia, Italy; 14 Otorhinolaryngology-Head and Neck Surgery, Department of Neuroscience, University of Padua, Padua, Italy; 15 Neuroradiology, Department of Translational Research On New Technologies in Medicine and Surgery, University of Pisa, Pisa, Italy; 16 Neuroradiology, Department of Medical and Surgical Specialties, Radiological Sciences and Public Health, University of Brescia, Brescia, Italy; 17 Pathology Unit, Department of Molecular and Translational Medicine, University of Brescia, Brescia, Italy; 18 Neurosurgery, Fondazione Policlinico Universitario Agostino Gemelli, IRCCS, Rome, Italy; 19 Neurosurgery, Dipartimento di Neuroscienze, Organi di Senso e Torace, Università Cattolica del Sacro Cuore, Rome, Italy; 20 Neurosurgery Dipartimento di Neuroscienze, Organi di Senso e Torace, Dipartimento di Neuroscienze, Fondazione Policlinico Universitario A. Gemelli, IRCCS, Università Cattolica del Sacro Cuore, Largo Agostino Gemelli, 8, Rome, 00168 Italy

**Keywords:** ACTH hyperplasia, Cushing's disease, Hypercortisolism, MRI negative, PitNET, Pituitary exploration

## Abstract

Cushing’s Disease (CD) poses diagnostic and therapeutic challenges, particularly when pituitary MRI is negative for a neuroendocrine tumor (PitNET). This study systematically analyzes literature data on the surgical outcomes of patients with MRI-negative CD and presents a novel single-center series. A systematic review and meta-analysis of PubMed, Scopus, and Cochrane databases (2000–2024) were performed. A retrospective analysis of patients with MRI-negative CD who underwent endoscopic pituitary exploration between 2014 and 2021 at the University of Brescia was conducted. A meta-analysis of 35 studies revealed significant heterogeneity across studies (I^2^ = 71.09%). The mean surgical disease remission rate of 70% (CI: 65–75%) in MRI-negative CD was significantly lower than in MRI-positive patients (82%; CI: 0.79–0.86). Eight studies reported ACTH hyperplasia in a total of 92 of 470 patients (19.6%). In the institutional series, 21 patients underwent 22 endoscopic pituitary explorations (mean follow-up: 72 months). Histology documented ACTH hyperplasia in 10/22 (45%), ACTH-PitNET in 9, and normal pituitary in 3. Inferior petrosal sinus sampling lateralization was predictive of pathology in 33% of patients. Early and late surgical remission (10/22 and 15/22 patients, respectively) were significantly associated with histology. Surgical complications included one CSF leak requiring reintervention, one case of transient diabetes insipidus, and two patients requiring substitutive therapy for the thyroid axis. About 30% of MRI-negative CD patients do not achieve surgical disease remission. ACTH hyperplasia might partially explain this high rate of failure. Endoscopic pituitary exploration remains a highly effective way to collect important histological data that should be recorded in a multicenter, prospective study.

## Introduction

Cushing’s disease is a rare disorder of hypercortisolemia, generally caused by an ACTH-secreting Pituitary Neuroendocrine Tumor (PitNET), and is associated with high morbidity and mortality [1]. Magnetic Resonance Imaging (MRI) is used to identify the PitNET but can be inconclusive in up to 64% of cases, even if dynamic and 3T MRI are used to improve the chance of identifying small microadenomas [2–5]. 

**Fig. 1:**
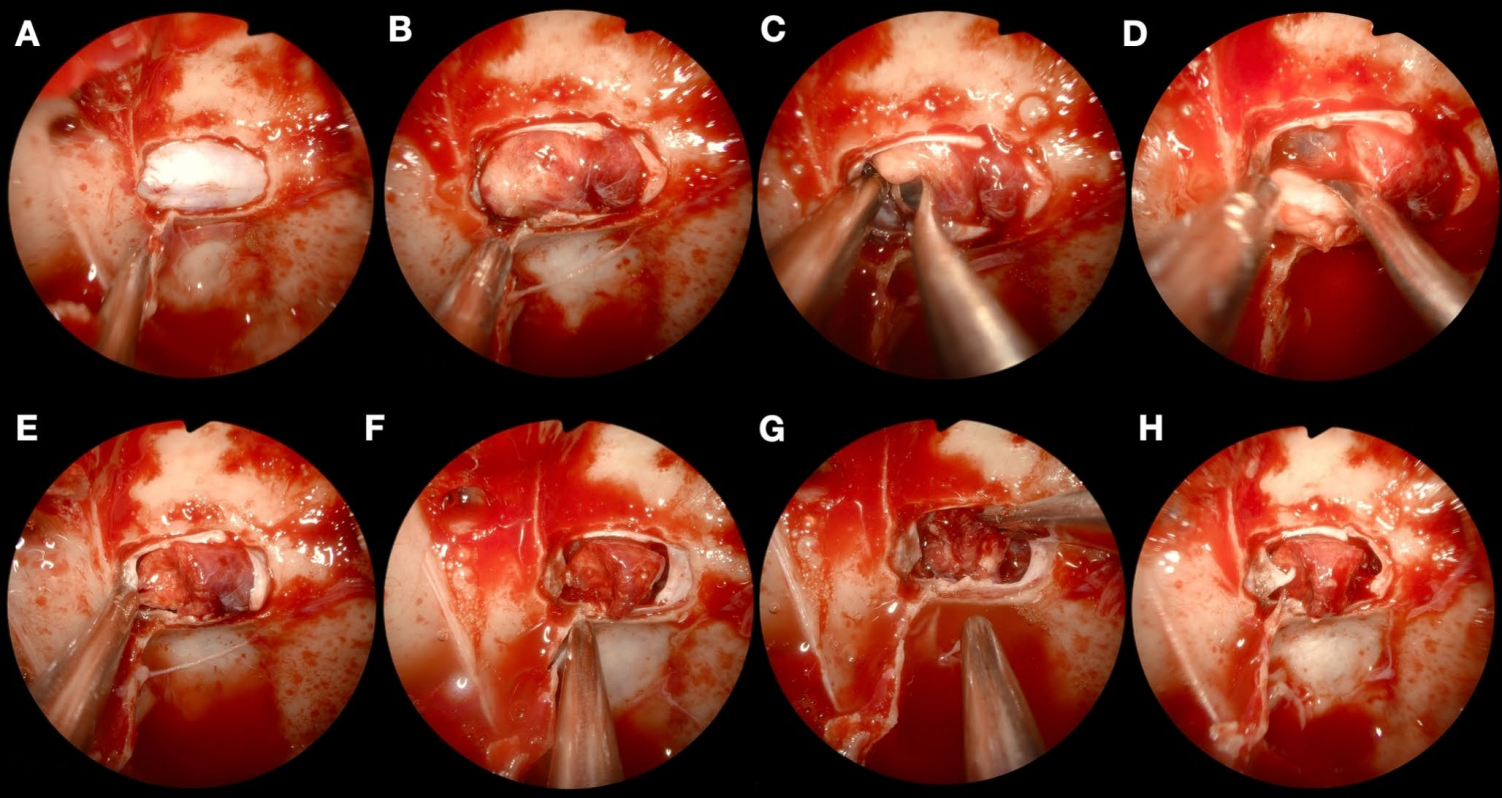
Steps of the endoscopic pituitary exploration. **A**: Opening the sellae protuberance and exposure of the periosteum; **B**: Cross incision of the periosteum and identification of the pituitary gland; **C**: Extra pituitary exploration on the right side; **D**: Superior pituitary exploration; **E**: Right pituitary exploration with removal of the third lateral of the gland; **F**: Left pituitary exploration with removal third contralateral; **G**: Median and posterior gland exploration; **H**: Final view with the preservation of the pituitary gland

Whether or not a PitNET is visible at MRI, when biochemical tests and Inferior Petrosal Sinus Sampling (IPSS) demonstrate central ACTH secretion, surgery should be suggested [3, 6–10]. While patients with visible PitNET at MRI undergo an adenomectomy using the pseudocapsule [11], patients with CD and normal or inconclusive MRI [10] undergo careful exploration of the pituitary gland (the so-called “pituitary exploration” – Fig. 1) through transsphenoidal surgery.

It is essential to collect and process each sample collected at surgery since serial sectioning may be necessary to characterize the underlying pathology [12]. Among the different scenarios, the pathologist can confirm no morphological abnormality, corticotroph hyperplasia, or ACTH PitNET [6]. Corticotroph hyperplasia is defined by the presence of expansion of ACTH-secreting cells with enlarged acini and a slight expansion of the reticulin network [13]. Although corticotroph hyperplasia represents a challenge both from diagnostic and management points of view, it can provide insights into the pathophysiological mechanisms underlying MRI-negative CD [13]. Data reported in the literature, however, remain sparse and limited.

In this study, we retrospectively analyzed 21 consecutive patients who underwent endoscopic pituitary exploration because of MRI-negative CD, at a single institution by the same surgical and pathology team. Furthermore, a systematic review of the literature with meta-analysis was performed to summarize and analyze the reported data on CD patients with negative MRI.

## Materials and methods

### Systematic literature review with meta-analysis

#### Search strategy

A systematic review was performed by searching articles published between January 1, 2000, and July 1, 2024, on PubMed, Scopus, and Cochrane with the following keywords: Cushing Disease AND negative MRI; Cushing Disease AND hyperplasia; ACTH adenoma AND negative MRI; Cushing Disease AND transsphenoidal surgery AND remission. The systematic review is reported according to the Preferred Reporting Items for Systematic Reviews and Meta-Analyses (PRISMA) guidelines [14]. 

#### Inclusion and exclusion criteria

The inclusion criteria were as follows: (1) articles published in English between January 1, 2000, and July 1, 2024; (2) randomized controlled trials and observational studies; (3) patients with negative brain or sellar MRI; (4) patients with positive sellar or brain MRI; (5) CD patients undergoing transsphenoidal (microscopic or endoscopic) surgery; (6) studies that measured remission data as an outcome.

The exclusion criteria were: (1) ACTH-adenomas in pediatric patients (under 18 years old); (2) low-field MRI (i.e., below 1 Tesla); (3) literature reviews lacking new data; (4) conference abstracts, letters to the editors, and comments; (5) studies reporting remission rates of either 0% or 100% (statistical variance = 0).

#### Quality assessment and data extraction

Articles were imported into the reference management software Zotero (version 5.0.92), and duplicates were removed manually. The titles and abstracts of the records retrieved were examined by two authors (A.M. and F.G.), and irrelevant citations were excluded. After randomly selecting a sample of the citations excluded, 20% were double-checked by another author (F.D.). Any disagreements were resolved through discussions among the reviewers. For each study, the following information was extracted: (1) authors and year of publication; (2) number of patients with negative and positive MRIs included in the study; (3) number of patients who underwent IPSS; (4) total remission of patients with negative and positive MRI.

#### Statistical methods

The meta-analyses were performed using the statistical software R version 3.6.3 (R Foundation for Statistical Computing, Vienna, Austria) [15] and the meta-packages [16]. Cochran’s Q-test and I2 statistics were applied to assess heterogeneity in the studies included in the meta-analysis. No heterogeneity was considered for *p* > 0.05 and I2 < 20%. A random-effect model was therefore adopted for the meta-analysis, with restricted maximum likelihood (REML) to estimate the heterogeneity variance.

### Institutional case series

The study included all patients affected by CD and with negative sellar MRI who underwent surgical pituitary exploration at the Neurosurgery Unit of the University of Brescia between February 2014 and July 2021 by the senior author (F.D.). The Institutional Review Board at the University of Brescia, Italy (NP 5071 – OMCPitNET) approved the study. In accordance with national regulations, informed consent was obtained from each patient for use of personal data.

The study inclusion criteria were as follows: (1) patients diagnosed with CD; (2) patients with preoperative imaging that included at least one negative brain MRI for pituitary pathologies; (3) patients undergoing surgery for primary or recurrent disease at the Neurosurgery Unit of Spedali Civili in Brescia.

Exclusion criteria included: (1) absence of complete, significant data (e.g., endocrinological data and follow-up); (2) patients with follow-up less than 3 months; (3) patients under 18 years of age or unable to provide consent.

#### Data collected

Demographic information, including the patient’s age and gender, clinical data, endocrinological and radiological investigations, surgical procedures and associated complications, histological results, and follow-up information (disease status and any adjuvant therapy), were collected retrospectively. Additionally, each patient completed a semi-structured questionnaire mainly focused on their current clinical status, including the presence or absence of cushingoid features and symptoms before and after surgery, any current or adjuvant therapy, and follow-up data, incorporating details about the most recent biochemical evaluation and MRI for CD.

In the presurgical work-up, IPSS was frequently used to confirm the pituitary origin of ACTH oversecretion after biochemical diagnosis of CD. ACTH ratios of the left and right inferior petrosal sinus (IPS) sample to the peripheral (P) venous blood sample were calculated (IPS: P). IPSS was deemed diagnostic for a pituitary source of ACTH production if the peak basal ratio (peak ratio before corticotropin-releasing hormone (CRH) administration) was greater than 1.4 or if the peak ratio after CRH administration was greater than 3. The inter-petrosal gradient ratio was considered predictive of lateralization if it was at least 1.4 before CRH administration and 3 after CRH administration.

#### Surgical exploration

All patients underwent a thorough pituitary exploration (Fig. 1) via an endoscopic transsphenoidal approach conducted by a surgical team led by the same neurosurgeon (F.D.), who had over 10 years of experience in transsphenoidal surgery and had performed more than 500 such procedures at the first pituitary exploration included in the study. Intraoperative findings were assessed and categorized into three groups: (1) no evidence of adenoma (PitNET); (2) suspicious findings for adenoma; (3) clear evidence of adenoma.

Various endoscopes were used throughout the study period (SD- and HD-3D, and 2D-4 K [17, 18]).

#### Pathological examination

**Fig. 2:**
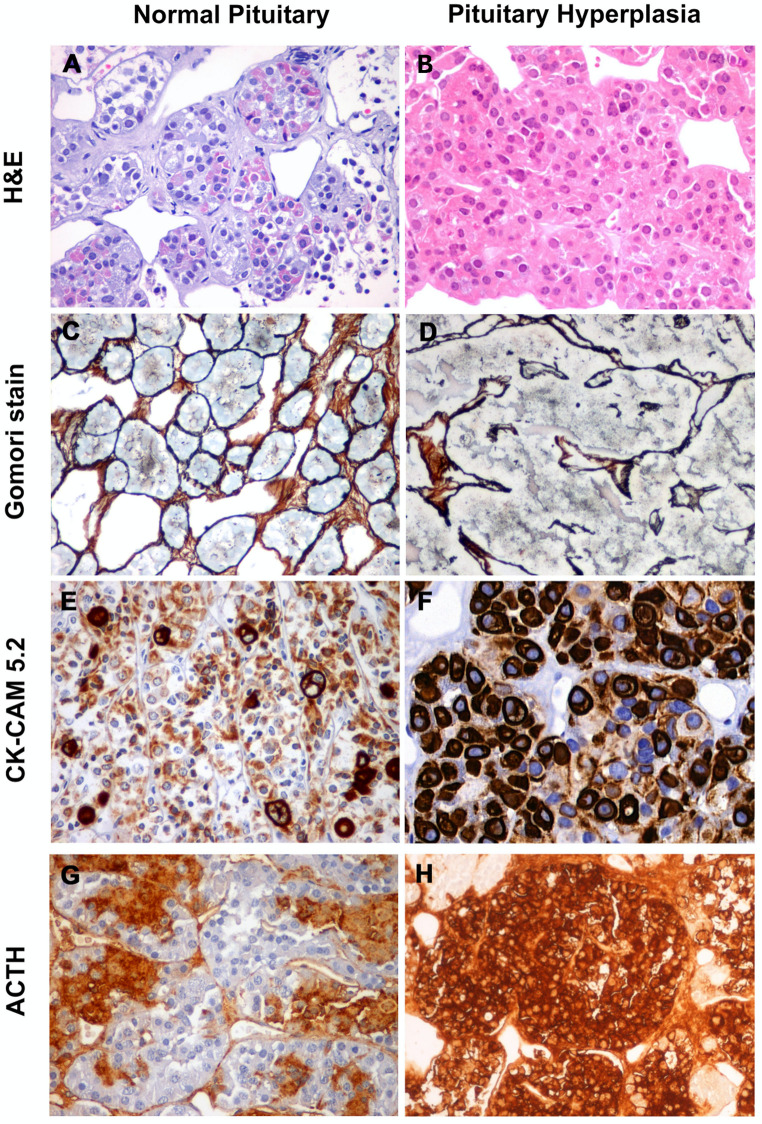
Representative images of normal pituitary tissue and pituitary hyperplasia. H&E staining shows a normal pituitary gland composed of small acini with both acidophils and basophils cells with fenestrated capillaries and with preserved reticulin architecture (**A-C**). Pituitary hyperplasia shows expanded acini with a more homogeneous staining and a slight expansion of the reticulum network (**B-D**). Compared to normal pituitary tissue (**E**), pituitary hyperplasia shows diffuse immunoreactivity for low-weight cytokeratins (CAM 5.2) with frequent Crooke cell changes, consisting of densely eosinophilic strongly immunoreactive hyaline material (**F**). Normal pituitary tissue shows focal immunoreactivity for ACTH (**G**), while pituitary hyperplasia exhibits homogeneous immunopositivity for ACTH (**H**). Expanded acinar architecture, Crooke cell changes and preponderance of ACTH staining within these areas represents important keys to make the diagnosis of hyperplasia

Tissue samples for histological analysis were systematically obtained during surgery and reviewed by the same pathologist (P.L.P.). Routine histological examination was performed on Formalin-fixed Paraffin-embedded (FFPE) tissue sections. Immunophenotypical analysis was conducted using Hematoxylin and Eosin (H&E) staining and immunohistochemistry, including expression of synaptophysin, low molecular weight cytokeratin, p53, hormonal markers, and assessment of Ki-67 proliferative index. Silver staining techniques were used to evaluate the preservation or loss of the reticulin network. Histologically, corticotroph hyperplasia was defined as the expansion of ACTH-secreting cells with focal or diffuse enlargement of pituitary acini and expansion of the reticulin network (Fig. 2) [19, 20]. 

#### Post-operative data

Remission was defined as the regression in the signs and symptoms of CD, with serum cortisol levels in the normal range or lower, necessitating hydrocortisone replacement therapy for at least 6 months following surgery. Data regarding substitution therapy were gathered during the last follow-up visit and through telephone interviews. Recurrence was defined as the occurrence of CD after initial biochemical remission.

### Statistical analysis

Data were analyzed using descriptive statistics and compared with the Fisher exact test for qualitative variables and the Wilcoxon test for quantitative variables (Kruskal for more than two groups). Meta-analysis was performed with the package metafor in the R statistical software and Test of Moderators was used to compare remission rates in the different groups.

## Results

### Systematic literature review with meta-analysis

**Fig. 3:**
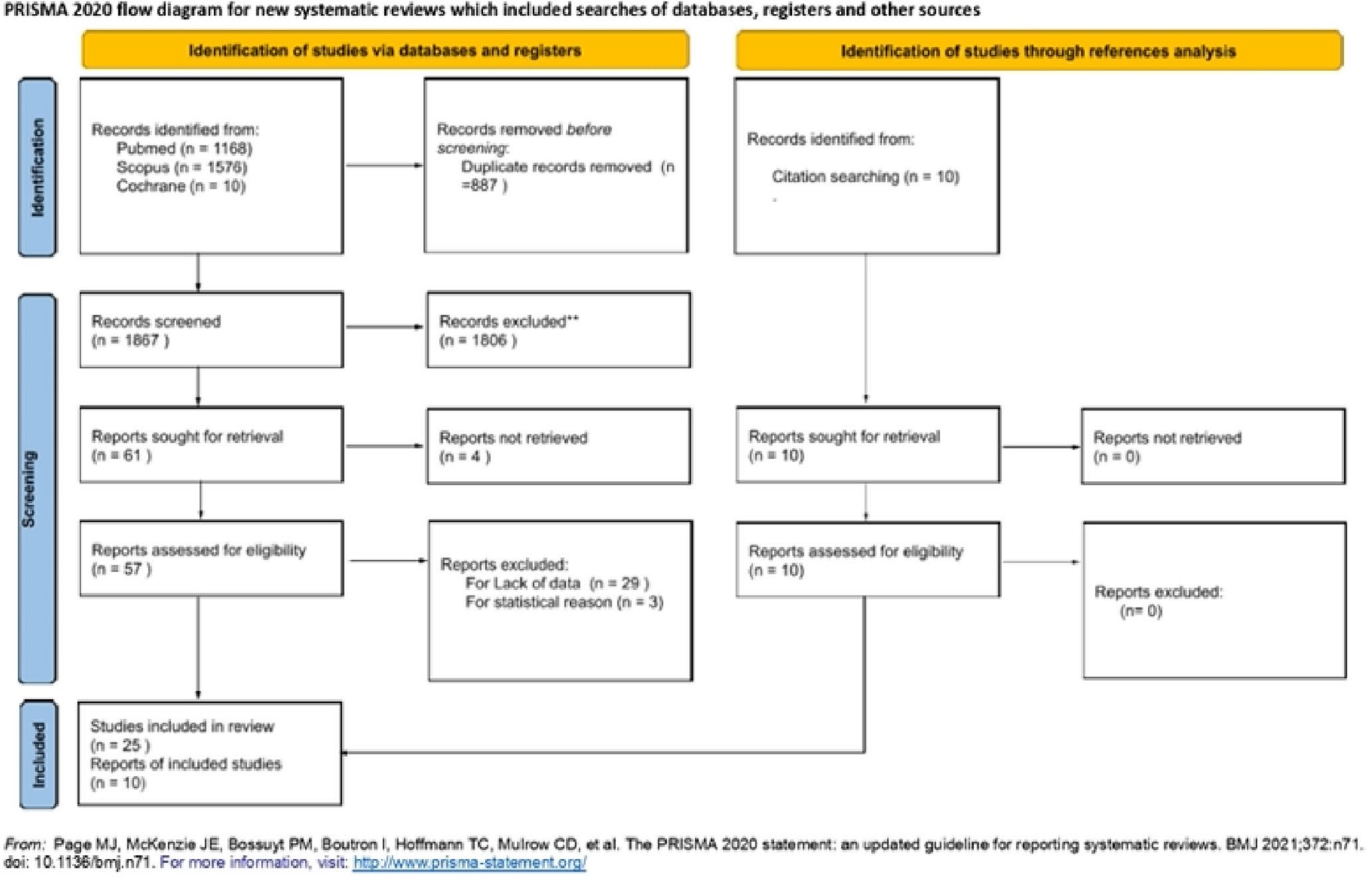
Literature review according to the PRISMA statement

The initial literature search yielded 2,754 articles, from which 887 duplicate records were removed. After reviewing the abstracts and titles, 1,806 articles were excluded. Of the 61 articles selected for full-text screening, 4 records could not be retrieved, while 57 met the inclusion criteria. Thirty-two articles were excluded due to a lack of data (29 articles) or statistical reasons (i.e., outcomes reported as 0 or 100% in three articles). An additional 10 records were identified through citation searching and assessed for eligibility. A total of 35 studies [1–3, 6, 9, 10, 21–49] were included in the final literature review (Fig. 3) [14]. 

### See text for further details

#### Heterogeneity and sensitivity analysis

**Fig. 4:**
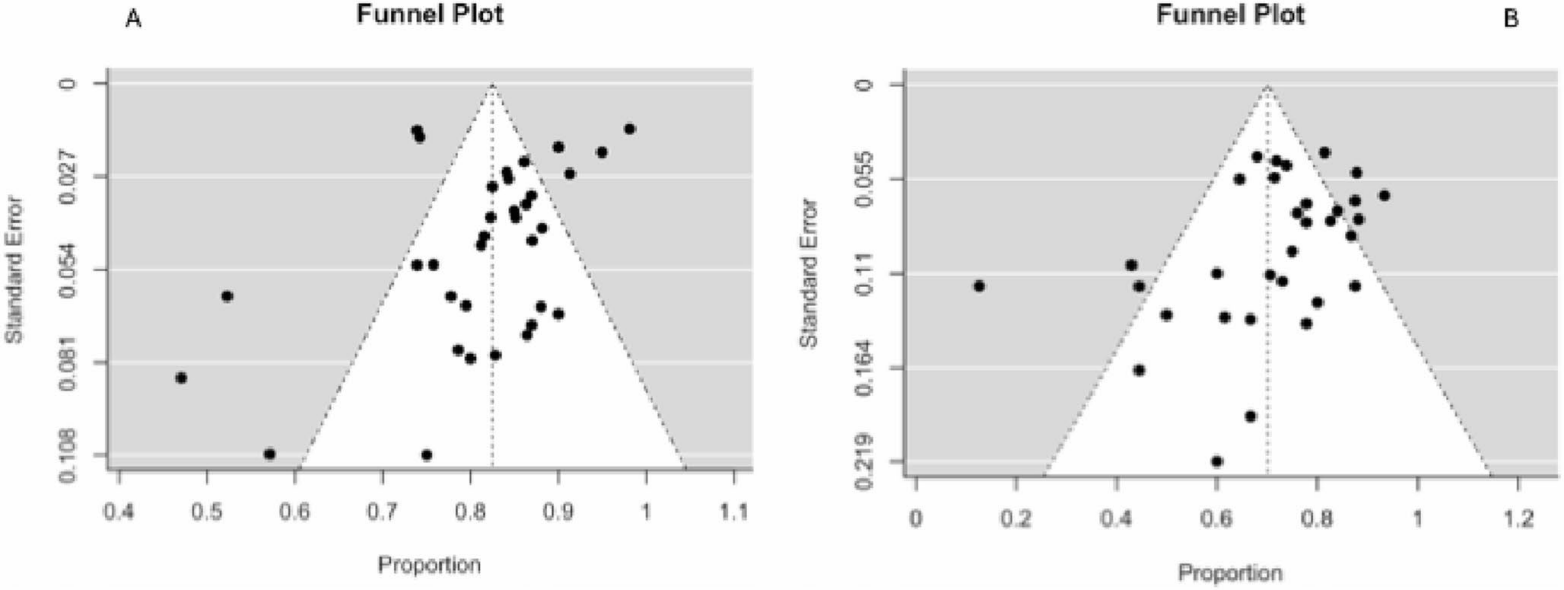
Funnel plot of the meta-analysis of published studies. MRI positive group (**A**) and MRI negative group (**B**). See text for further details

Significant heterogeneity was observed in the MRI-positive group (I^2^ = 86.21% *p* < 0.0001; estimate = 0.8249; standard error for estimate = 0.0156), while a lower heterogeneity was identified in the MRI-negative group (I^2^ = 71.09% *p* < 0.001; estimate = 0.7010; standard error for estimate = 0.0271) (Fig. 4). Overall, limited publication bias was observed only in the MRI-negative group, as indicated by Egger’s regression test (MRI-positive group: *p* = 0.0648, MRI- negative group: *p* = 0.0062), and limited number of data points outside the 95%. confidence interval (dotted line).

#### Demographics and radiological features

A total of 1,131 patients with CD and negative MRI underwent exploration of the pituitary gland. Only 15 authors^4^ [2, 3, 6, 9, 10, 21, 22, 26–28, 36, 38, 44, 48, 49] specified the magnetic field strength; in te10n centers, a 3T MRI was performed exclusively or after a 1.5T MRI exam.

#### Preoperative diagnostic evaluation

Seventeen studies [1–3, 6, 21, 22, 25–27, 30, 33, 34, 37, 38, 40, 41, 44] described 677 patients who underwent IPSS. IPSS tested positive for lateralization in 611 cases.

#### Operative procedures, outcomes, and findings

**Fig. 5:**
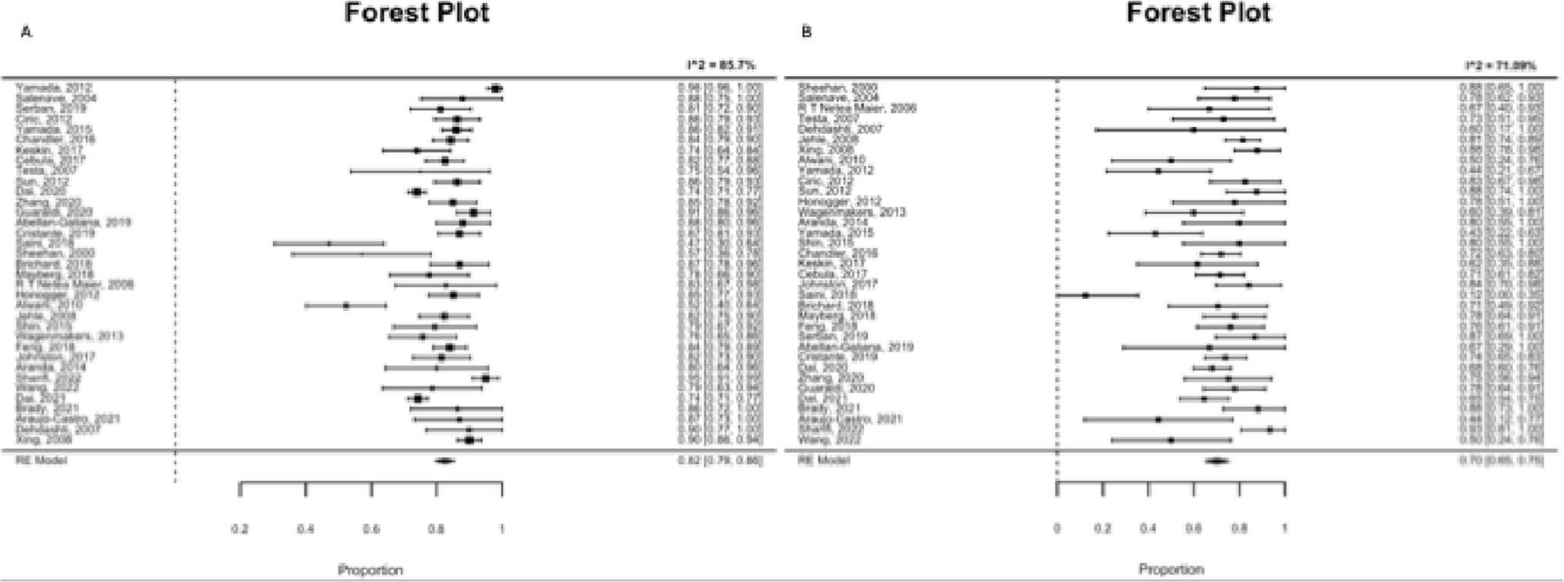
Forest plot of the remission rate in the positive and negative MRI groups. MRI positive group (**A**) and MRI negative group (**B**). See text for further details

The meta-analysis indicated a lower remission rate in the MRI-negative group (RR 0.70, CI 95% 0.65–0.75) compared to the MRI-positive group (RR 0.82, CI 95% 0.79–0.86) (Fig. 5). In 224 cases, no adenoma was visualized during surgical pituitary exploration. A PitNET was recognized and removed in 519 patients.

Eight studies [6, 21, 26, 27, 35, 37, 44, 46] reported that 92 of 470 (19.6%) MRI-negative patients had evidence of ACTH hyperplasia at histological examination.

### Institutional case series

Patient data from the institutional series are summarized in Table 1. A total of 21 patients (7 males and 14 females) were included with a mean age of 47.6 years (range 18 to 74 years). All were referred for surgery with a biochemical diagnosis of CD. IPSS was conducted in 15 of 22 cases, and detailed data were available for 13 patients. IPSS was positive in 12 (only after basal: 6; only after CRF: 2; in both basal and CRF: 4) and negative in one. IPSS demonstrated lateralization on the left side in 5 of 12 patients and on the right side in 7 (Table 1). IPSS lateralization aligned with the location of the pathology on the histopathology specimens in 33% (4 of 12) of patients (Table 1).

All patients underwent pituitary exploration using an endoscopic transsphenoidal approach. A total of 22 procedures were carried out, including one patient who underwent two explorations (patients 9 and 14 in Table 1). Intraoperatively, the surgical team clearly identified pathological tissue in 4 patients, noted questionable tissue in 16, and recorded no pathological tissue in 2 patients.

Histological examination documented an ACTH PitNET in 9 cases (40.9%), hyperplasia in 10 (45.5%), and no pathological tissue in 3 cases (13.6%) (Table 1). Of the 22 cases, 10 (45%) experienced early surgical remission, and 15 of 22 cases (68.2%) had achieved biochemical remission at the last follow-up (Tables 1 and 2).

Early surgical remission and histological findings were associated with the last remission (Table 2). The group of patients with histological confirmation of a PitNET experienced complete remission at the last follow-up (9 patients, 100%), while only 6 of 10 patients (60%) with hyperplasia achieved remission. Among these, 2 patients had early and persistent surgical remission, while 4 attained disease remission only through adjuvant therapy, including medical treatment, adrenalectomy, or both (Table 1). Conversely, the 3 patients with histological confirmation of no pathological tissue did not achieve remission at the last follow-up, even with medical therapies (Tables 1 and 3).

Early surgical remission is associated with the latest disease status (Table 3). All 10 patients who achieved early surgical remission remained in remission at the last follow-up (mean: 72 months; range: 27–111). Long-term disease remission with adjuvant therapies was attained in only 5 of 12 patients who did not experience early surgical remission.

Surgical complications included one patient who required reintervention due to a CSF leak without meningitis. One patient experienced transient diabetes insipidus, while 2 patients required substitutive therapy for the thyroid axis (one had undergone 2 explorations).

**Table 1:** Patient data. Demographic and clinical data of 21 patients (22 surgical procedures)

#	Age/ gender	IPSS	IPSS Side	Intraop finding	Intraop side	Histology	Path. Side	Early remission (3 months)	Adjuvant therapy	Long - term remission	FU (m.)
1	38/F	Pos (B)	L	PitNET	Bilat	PitNET	Bilat	Yes	None	Yes	111
2	51/M	Pos (B + C)	R	Doubt	L	PitNET	Bilat	Yes	None	Yes	110
3	18/F	Pos (B)	R	Doubt	L	Normal	-	No	Med	No	110
4	46/F	Neg	-	Doubt	L	Normal	-	No	Med	No	104
5	59/F	Pos (B + C)	R	Doubt	R	PitNET	R	Yes	None	Yes	103
6	30/M	Pos (B + C)	L	Doubt	L	Hyper	L	No	Med, ADX	Yes	101
7	53/F	No	-	Doubt	Bilat	Hyper	Bilat	No	Med	No	92
8	41/M	Pos (NA)	NA	No PitNET	Bilat	Hyper	Bilat	No	Med	Yes	88
9	33/F	Pos (NA)	NA	Doubt	Bilat	Hyper	Bilat	No	Med	No	25
10	36/M	Pos (B)	L	Doubt	Bilat	Hyper	Bilat	Yes	None	Yes	87
11	59/M	Pos (C)	L	Doubt	R	PitNET	Bilat	No	Med, ADX	Yes	87
12	71/F	Pos (B)	R	Doubt	R	Hyper	R	No	Med	No	79
13	56/F	No	-	Doubt	L	Normal Pit	-	No	Med, RT	No	65
14	36/F	No	-	PitNET	Bilat	Hyper	Bilat	No	ADX	Yes	60
15	60/F	No	-	Doubt	R	Hyper	Bilat	No	ADX	Yes	52
16	53/F	Pos (B)	R	Doubt	R	Hyper	R	Yes	None	Yes	46
17	42/M	No	-	PitNET	R	PitNET	R	Yes	None	Yes	41
18	30/F	No	-	Doubt	R	PitNET	R	Yes	None	Yes	36
19	45/F	Pos (C)	R	Doubt	L	PitNET	L	Yes	None	Yes	34
20	74/F	Pos (B + C)	R	No PitNET	-	Hyper	Bilat	No	Med	No	30
21	54/M	No	-	Doubt	L	PitNET	L	Yes	None	Yes	29
22	63/F	Pos (B)	L	PitNET	R	PitNET	R	Yes	None	Yes	27

Abbreviations: ADX, adrenalectomy; B, at baseline evaluation; Bilat, bilateral; C, after CRH; F, female; FU, follow-up; IPSS, Inferior Petrosal Sinus Sampling; Intraop, intraoperative; M, male; m., months; L, left; Med, Medical Therapy; N, number; NA, Not available; Neg, negative; No, not performed; Path., pathology; Pit, pituitary gland; PitNET, Pituitary Neuro-Endocrine Tumor (adenoma); Pos, positive; R, right; RT, radiotherapy; y, years; -, data is not applicable

**Table 2:** Early surgical remission. Ten of 22 cases obtained early surgical remission

	Disease persistence (*N* = 12)	Remission (*N* = 10)	Total (*N* = 22)	*p* value
Histology				0.002
PitNET	1 (8.3%)	8 (80.0%)	9 (40.9%)	
Hyperplasia	8 (66.7%)	2 (20.0%)	10 (45.5%)	
Normal tissue	3 (25.0%)	0 (0.0%)	3 (13.6%)	
**Adjuvant therapy**				**< 0.001**
No	2 (16.7%)	10 (100.0%)	12 (54.5%)	
Yes	10 (83.3%)	0 (0.0%)	10 (45.5%)	

**Table 3:** Remission at the last follow-up. Remission at the latest follow-up was achieved in 15 of 22 cases (14/21 patients)

	Disease persistence (*N* = 7)	Remission (*N* = 15)	Total (*N* = 22)	*p* value
Histology				0.003
PitNET	0 (0.0%)	9 (60.0%)	9 (40.9%)	
Hyperplasia	4 (57.1%)	6 (40.0%)	10 (45.5%)	
Normal tissue	3 (42.9%)	0 (0.0%)	3 (13.6%)	
**Adjuvant therapy**				**< 0.001**
No	0 (0.0%)	12 (80.0%)	12 (54.5%)	
Yes	7 (100.0%)	3 (20.0%)	10 (45.5%)	
**Early surgical remission**				**0.005**
No	7 (100.0%)	5 (33.3%)	12 (54.5%)	
Yes	0 (0.0%)	10 (66.7%)	10 (45.5%)	

## Discussion

CD is a severe condition that presents various diagnostic and therapeutic challenges. Diagnostic challenges include the endocrinological and radiological assessments used to diagnose CD and document the ACTH PitNET. These challenges are even more complex when MRI is inconclusive or normal. A rigorous, non-dogmatic approach that analyzes potential pitfalls at each diagnostic and therapeutic phase is necessary.

### The presurgical diagnostic challenge

A thorough clinical and biochemical diagnosis process is essential for properly diagnosing CD and excluding pseudo-Cushing conditions. Pseudo-Cushing’s syndrome represents a complex clinical scenario characterized by mild-to-moderate ACTH-dependent hypercortisolism due to CRH and/or vasopressin hypothalamic secretion through the activation of various neural pathways [50]. Since a non-neoplastic etiology is recognized in Pseudo-Cushing syndrome, this condition has been redesignated as “non-neoplastic hypercortisolism” (NNH), being mainly due to neuropsychiatric disorders, alcohol abuse, insulin-resistant obesity, polycystic ovary syndrome, and end-stage kidney disease [50]. As suggested by the last consensus [51], NNH should be excluded in all patients considered at risk for CD and with abnormal late-night salivary cortisol (in at least two determinations), 24-hour urinary free cortisol, and low-dose suppression test. Therefore, the investigation of clinical history, use of concomitant drugs, and screening for catabolic signs of hypercortisolism are mandatory to orient the diagnosis, together with biochemical tests. In patients with NNH, serum and urine cortisol, midnight cortisol, and low-dose suppression test are often abnormal, and the dexamethasone-suppressed corticotropin-releasing hormone (CRH) stimulation test has good sensitivity (91%) and specificity (82%) in discriminating CD from NNH [52]. 

Due to its retrospective design, our clinical study did not thoroughly investigate the endocrinological diagnosis of CD. Expert endocrinologists and neuroradiologists investigated all patients.

### Inferior petrosal sinus sampling

IPSS is currently recognized as a gold standard in the diagnosis of CD, with a sensitivity of approximately 92.1%–98.9% [45]. All our patients underwent CRH stimulation, though more recently other tests have been described [53, 54]. 

According to some studies, IPSS can be successfully applied to the preoperative evaluation of patients with presumed CD [21, 25].

In our series this was interpreted according to our predefined protocol using a basal IPS: peripheral ACTH ratio ≥ 1.4 and/or a post–CRH-stimulated ratio ≥ 3.0, with operative decisions primarily anchored to the stimulated gradient to preserve specificity in MRI-negative disease. Although conventional thresholds of ≥ 2.0 basally and ≥ 3.0 post-stimulation are widely referenced [55], multiple reports [56, 57] document center-level variability and support lower basal cut-offs to enhance sensitivity in appropriately selected patients. Early IPSS series and subsequent analyses acknowledge basal thresholds around 1.4–1.7 [58–60], and contemporary reviews note that receiver-operating-characteristic analyses in some cohorts favored basal cut-offs near 1.4 without compromising diagnostic performance when procedures are executed by experienced teams during active hypercortisolism [57, 61]. In line with these data and best-practice recommendations emphasizing the primacy of the stimulated gradient, our use of a basal threshold of 1.4 is intended to maximize sensitivity in biochemically active, MRI-negative CD.

Studies report that IPSS can guide intraoperative exploration of the pituitary gland [41, 45]. Prior large-scale studies [2, 62] reported positive predictive values for correct lateralization ranging between 61.34% [41] and 84.6% [45], suggesting that IPSS might help in identifying tumor lateralization. Testa et al. [26] underlined the low predictive value of IPSS in adenoma lateralization and that the surgical approach did not differ in patients who underwent IPSS from those who did not. Inaccurate lateralization has been attributed to asymmetrical venous drainage with shunting of blood toward the dominant side and to anatomical variation of the inferior petrosal sinus or to physiological fluctuations of ACTH secretion that can affect sampling results and catheter positioning [2, 45]. Data on venous anatomy could not be retrieved in our series. Interestingly, even when pathology was bilateral, IPSS showed lateralization according to recently suggested criteria.

Our series demonstrated low concordance between the lateralization suggested by IPSS and the side confirmed by surgery or histopathology: a positive correlation was found in only 4 of 12 (33%) patients. Indeed, all our patients underwent a systematic pituitary exploration, which started at the level of the most suspicious MRI finding or the lateralization sign of IPSS, but continued even if the team suspected that a PitNET might have been found. Given these limitations, thorough surgical exploration of the pituitary gland remains essential in MRI-negative patients regardless of IPSS-predicted lateralization to maximize chances of tumor identification and cure.

### The surgical challenge

Our systematic literature review shows that patients with CD and failed intraoperative adenoma visualization account for 30.5% of all those treated for MRI-negative CD, ranging from 17% to 63% [63, 64]. A mean early remission rate of 82% was documented in the MRI-positive group and 70% in the MRI-negative group; a wide variation among studies was also evident, with a confidence interval ranging from 79% to 86% and from 65% to 75%, respectively. In our clinical series, the early remission rate in patients with negative MRI was lower than in the literature (45%). The higher incidence of hyperplasia might explain the low remission rate: 10 cases of 22 (45%) had evidence of corticotroph hyperplasia.

### The pathological challenge

The histopathological diagnosis of corticotroph hyperplasia is still a debated issue, and clear-cut diagnostic criteria for its definition are still missing [13]. Thus, a diagnosis of corticotroph hyperplasia might be under-reported [19, 26]. However, evaluation of the reticulin network, along with immunohistochemistry for ACTH and low molecular weight cytokeratin, represents a useful tool in the differential diagnosis between normal pituitary tissue, hyperplasia, and PitNET [12, 19]. The systematic literature indeed documented a much lower incidence of ACTH hyperplasia, together with an extremely wide range among patients with negative MRI (2.4–77.6%) [44, 46].

In addition to histological analysis, no other diagnostic procedures are reliable enough to lead to the correct diagnosis of pituitary hyperplasia. In this cohort of patients, no significant correlation between histological findings (PitNET versus hyperplasia) and IPSS was found, even if the IPSS remains the gold standard in identifying the source of CD in patients with negative or equivocal MRI findings [65]. Moreover, intraoperative tissue is difficult to interpret even for an expert neurosurgeon, according to the recent definition of proficiency [66]. For instance, for the patient who was operated on twice in our series, there was the impression of seeing a PitNET both times, while histological analysis confirmed the diagnosis of hyperplasia. Disease remission was achieved only after bilateral adrenalectomy in this patient.

Pituitary hyperplasia is usually not associated with major modification of the pituitary gland, may be largely unrecognized on MRI analysis, and may also be difficult to recognize on histological analysis in small surgical specimens. Thus, performing partial hypophysectomy enables the collection of tissue samples with limited morbidity, allowing extensive histopathological analysis. Histopathological diagnosis is crucial, and investigation of the reticulin network and immunohistochemistry for ACTH and cytokeratins represent useful tools in the differential diagnosis between normal pituitary tissue, hyperplasia, and PitNET.

## Conclusion

Our findings must be interpreted considering certain limitations. Since the current study is retrospective, it is limited by the bias that complete data collection was precluded for all patients. Another limitation is the small sample size of the case series. Nonetheless, these limitations are compensated for by the systematic literature review and meta-analysis. Despite these limitations, our findings highlight that nearly 1 in 3 patients with CD and a negative pituitary MRI fail to achieve disease remission. This underscores the importance of thorough histopathological examination, which may reveal a high prevalence of ACTH hyperplasia that is not detected by imaging alone. When performed by experienced surgeons, pituitary exploration carries a low risk of complications and remains the definitive approach to remove an ACTH-PitNET if present. Moreover, it provides essential histological information that correlates with remission outcomes and can guide subsequent therapeutic decisions in cases where the disease persists. Overall, acknowledging the present limitations in the pre- and post-operative diagnostic phases of CD is important to plan future multidisciplinary, multicenter, systematic studies to further advance the diagnosis and management of patients with CD and negative MRI.

## Data Availability

All data that support the findings of this study are included in the published article.

## Abbreviations

ACTH: Adrenocorticotropic Hormone
CD: Cushing’s Disease
CRF: Corticotropin Releasing Factor
CRH: Corticotropin Releasing Hormone
CSF: CerebroSpinal Fluid
FFPE: Formalin-fixed Paraffin-embedded
H&E: Hematoxylin and Eosin
IPS: Inferior Petrosal Sinus
IPSS: Inferior Petrosal Sinus Sampling
MRI: Magnetic Resonance Imaging
NNH: Non-Neoplastic Hypercortisolism
P: Peripheral
PitNET: Pituitary Neuro-Endocrine Tumor
PRISMA: Preferred Reporting Items for Systematic Reviews and Meta-Analyses
